# Supplementary material for: Record-breaking Greenland ice sheet melt events under recent and future climate
Source: Nat Commun. 2026 Feb 11;17:3605. doi: 10.1038/s41467-026-69543-5 (PMC13096336; doi:10.1038/s41467-026-69543-5)
Supplement: Supplementary file 1 — Supplementary Information [file 41467_2026_69543_MOESM1_ESM.pdf]

Supplementary material

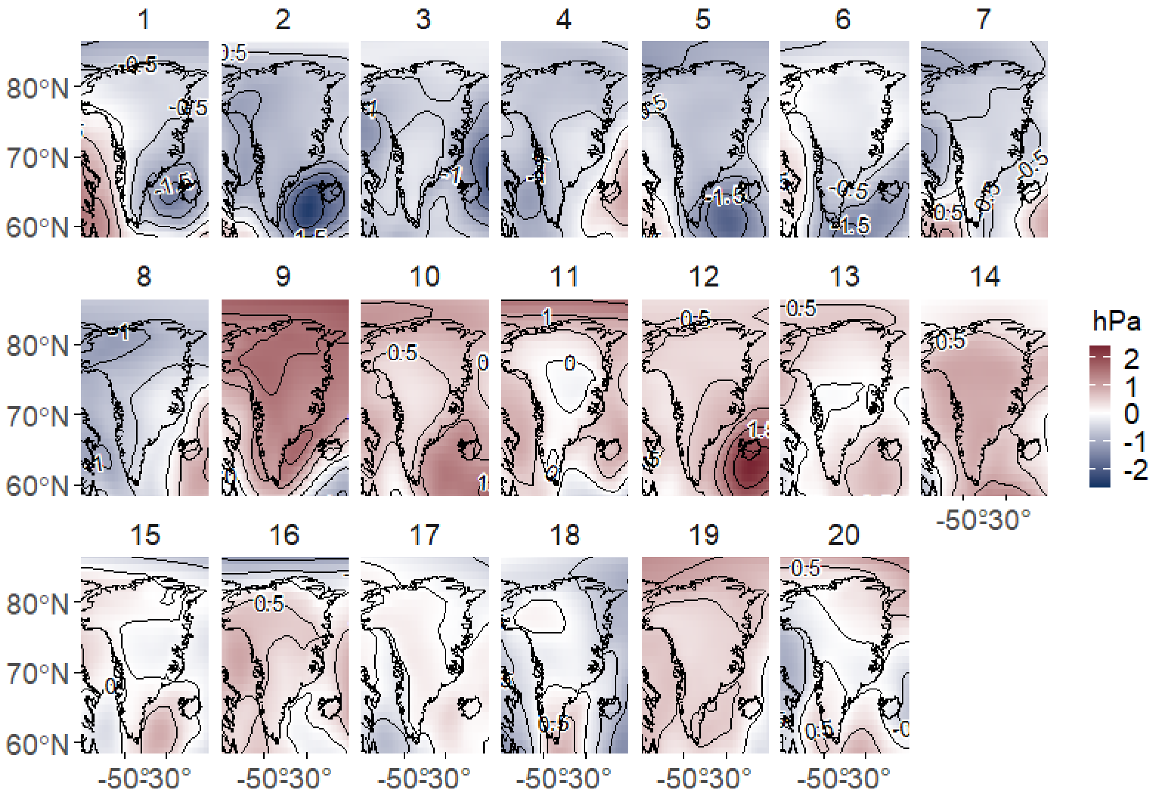

Figure S1. Spatial average anomalies of SLP (hPa) grouped by Circulation Weather Type (CWT). Anomalies are calculated by subtracting the average SLP from the average SLP for each CWT and at grid level from July and August (1950-2023).

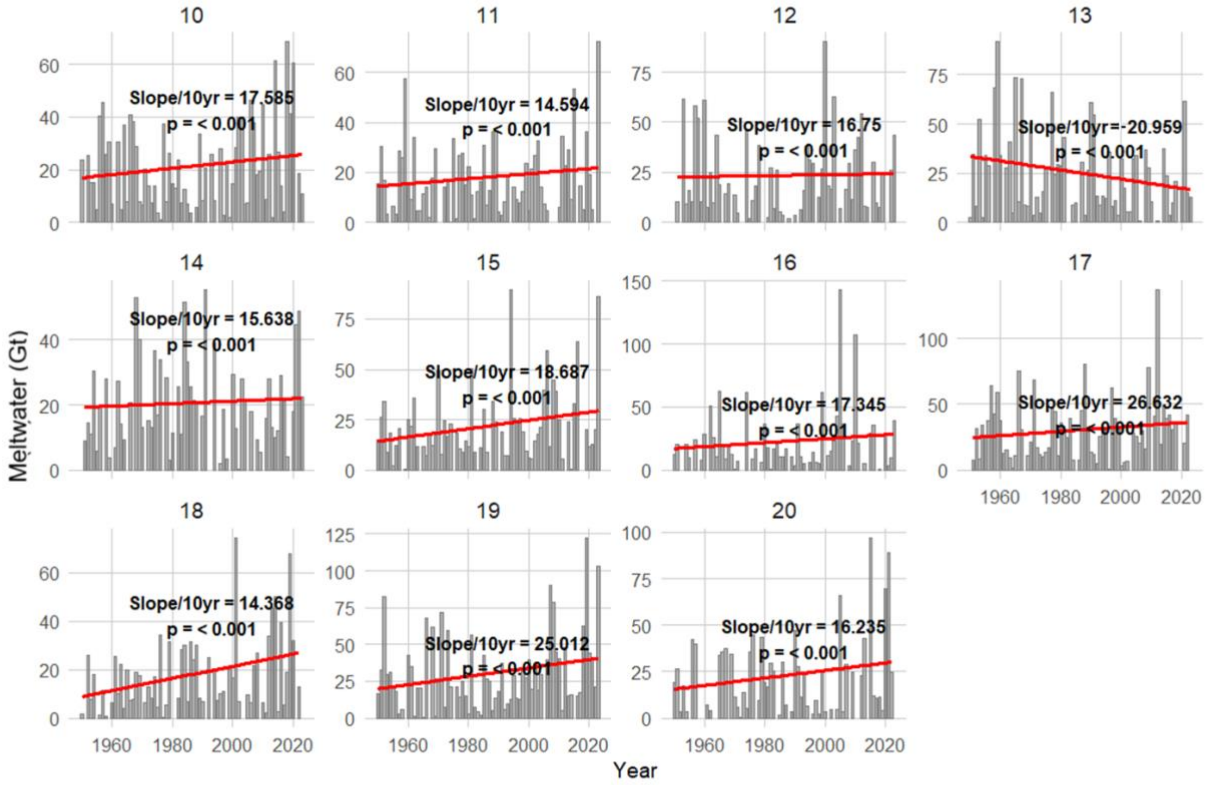

Figure S2. Temporal evolution of average meltwater accumulated and associated with anticyclonic CWTs. Red lines indicate linear trends for the 1950–2023 period, with slope values (per decade) and corresponding p-values shown.

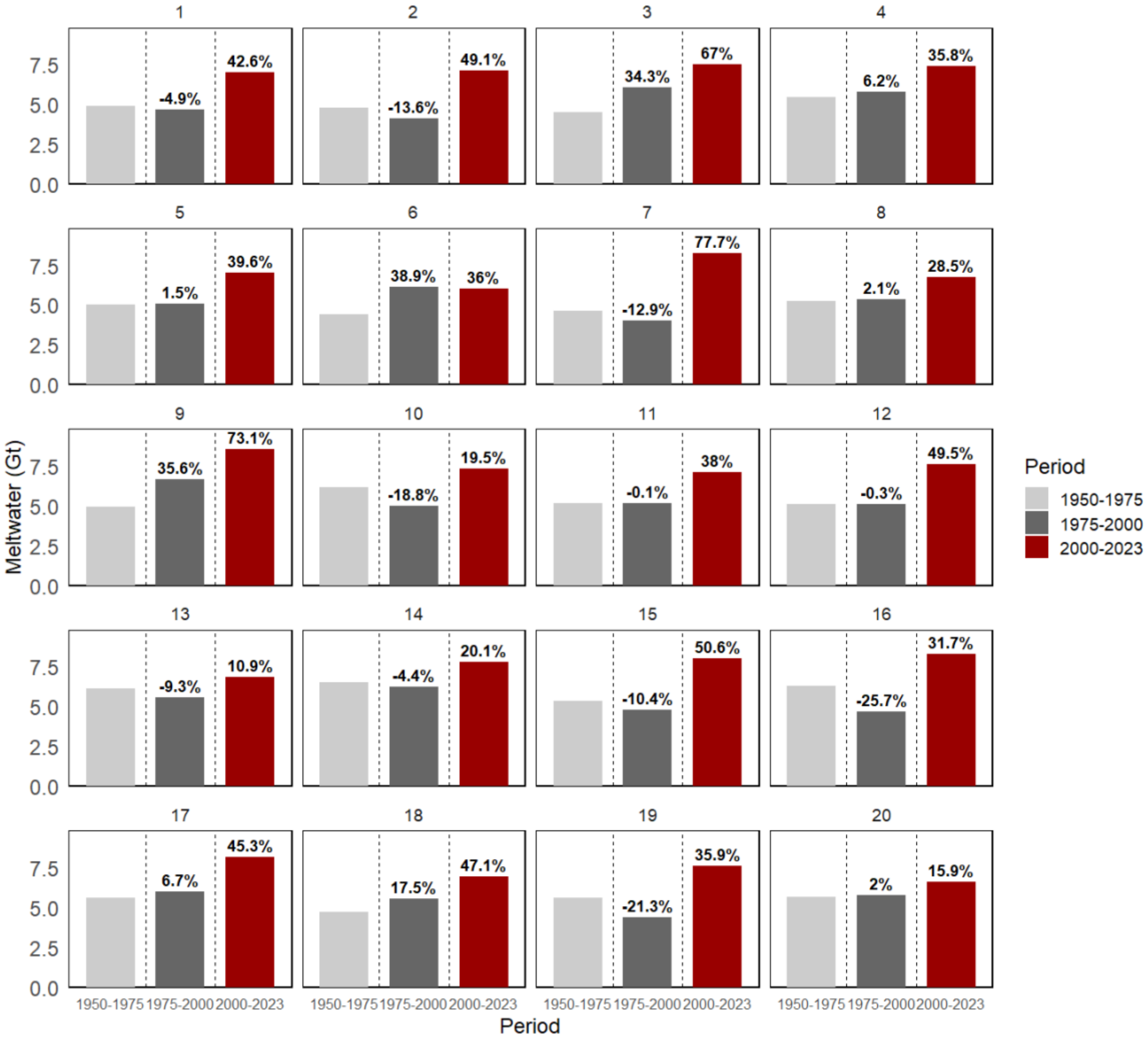

Figure S3. Average meltwater during the same CWT across different temporal periods. Annotations indicate anomalies relative to the CWT-specific average for the 1950–1975 period, a time only marginally influenced by climate change.

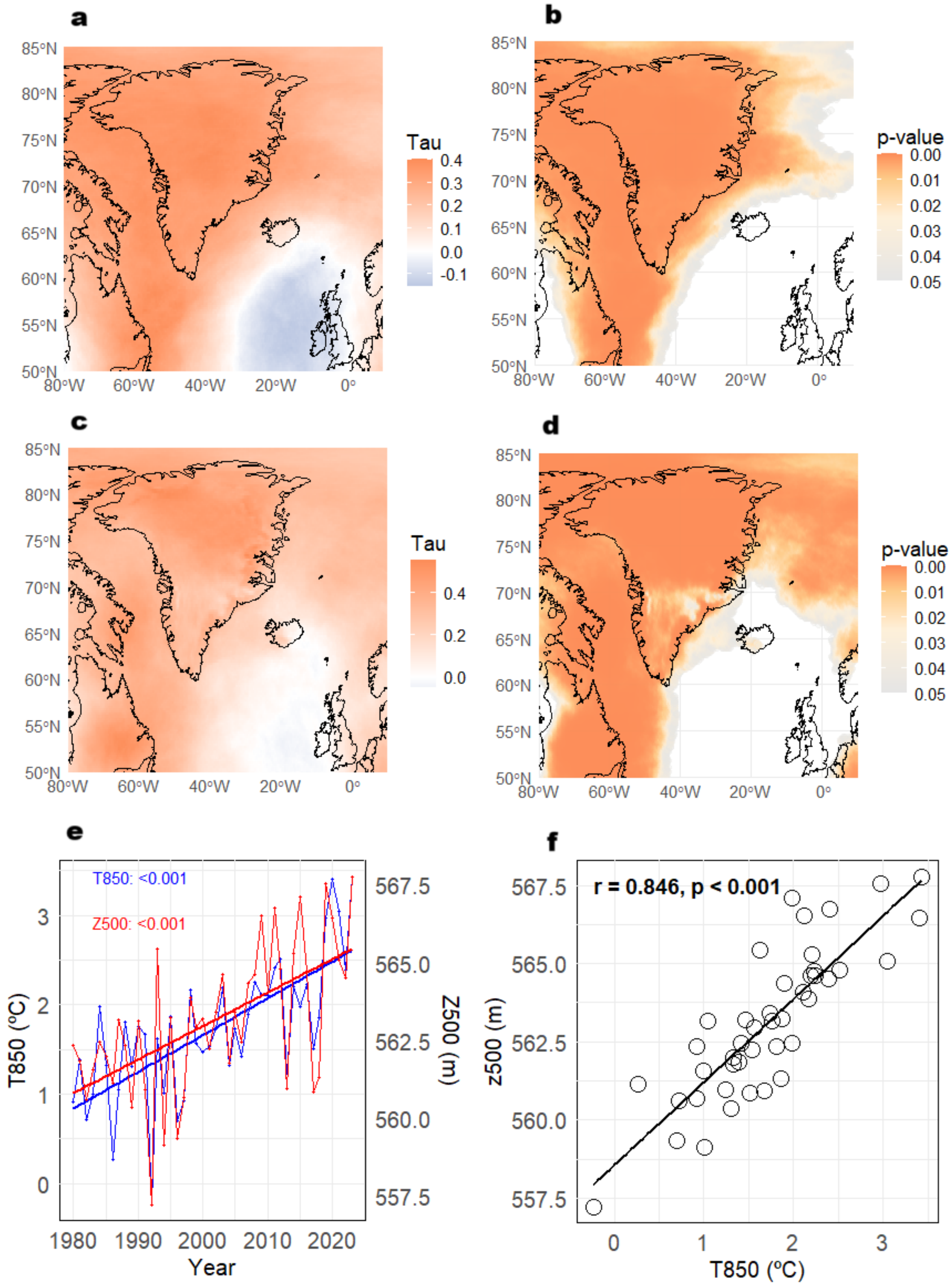

Figure S4. Mann-Kendall trend analysis for geopotential height at 500 hPa (Z500, m) and air temperature at 850 hPa (T850, °C) for July and August, 1980–2023. Panels (a) and (b) show the Mann-Kendall tau and p-value for Z500, respectively. Panels (c) and (d) show the same for T850. Temporal evolution of T850 (left y-axis) and Z500 (right y-axis) (e). Linear relationship between Z500 (m) and T850 (°C) (f). In some high-elevation areas of the ice sheet, the 850 hPa pressure level is below the surface. ERA5 provides values at this level via interpolation, but these do not correspond to a physical atmospheric layer. These values are retained for completeness but should be interpreted with caution.

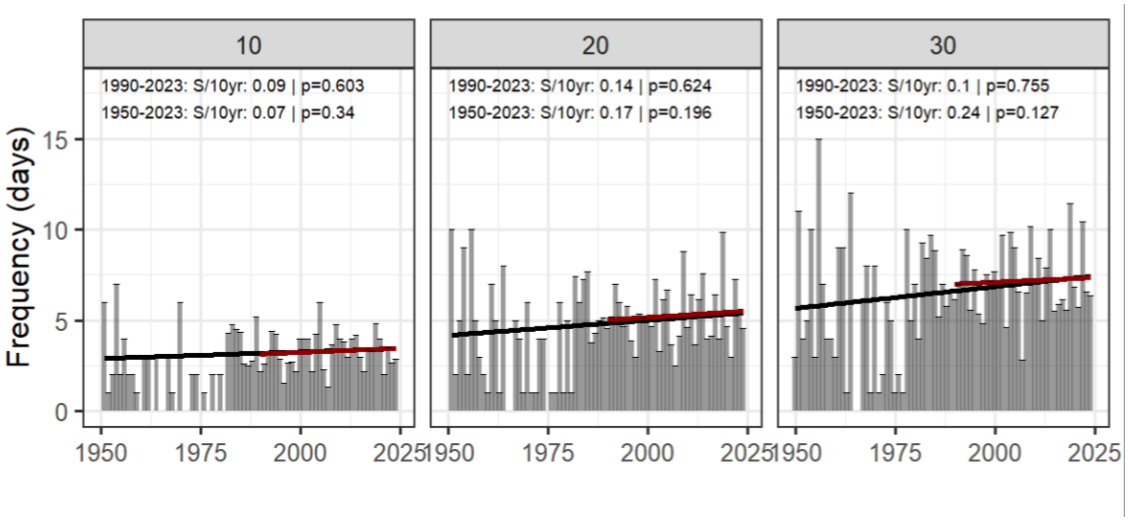

Figure S5. Temporal evolution of the July and August average frequency (days) of flow analogues corresponding to the top 10 extreme melting events. Black and red lines indicate linear trends for the periods 1950–2023 and 1990–2023, respectively, with slope values (per decade) and p-values annotated.

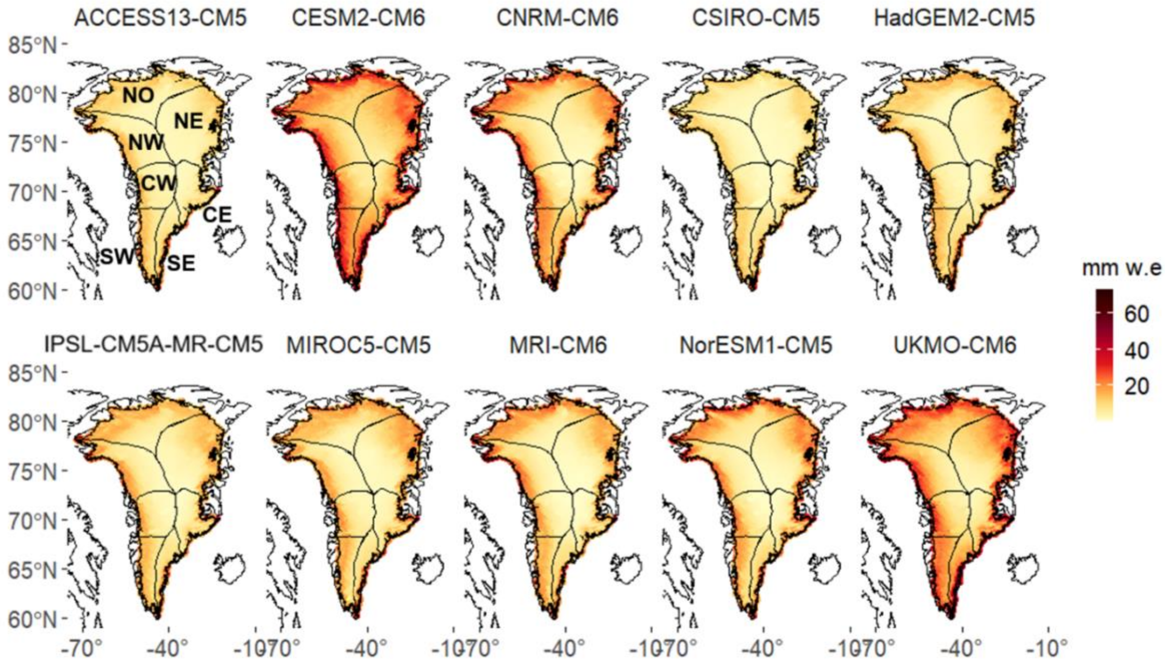

Figure S6. Average monthly 95th percentile of summer melt over the GrIS during July and August for the period 2090–2100. The suffix -CM5 refers to CMIP5 models, while -CM6 refers to CMIP6 models.

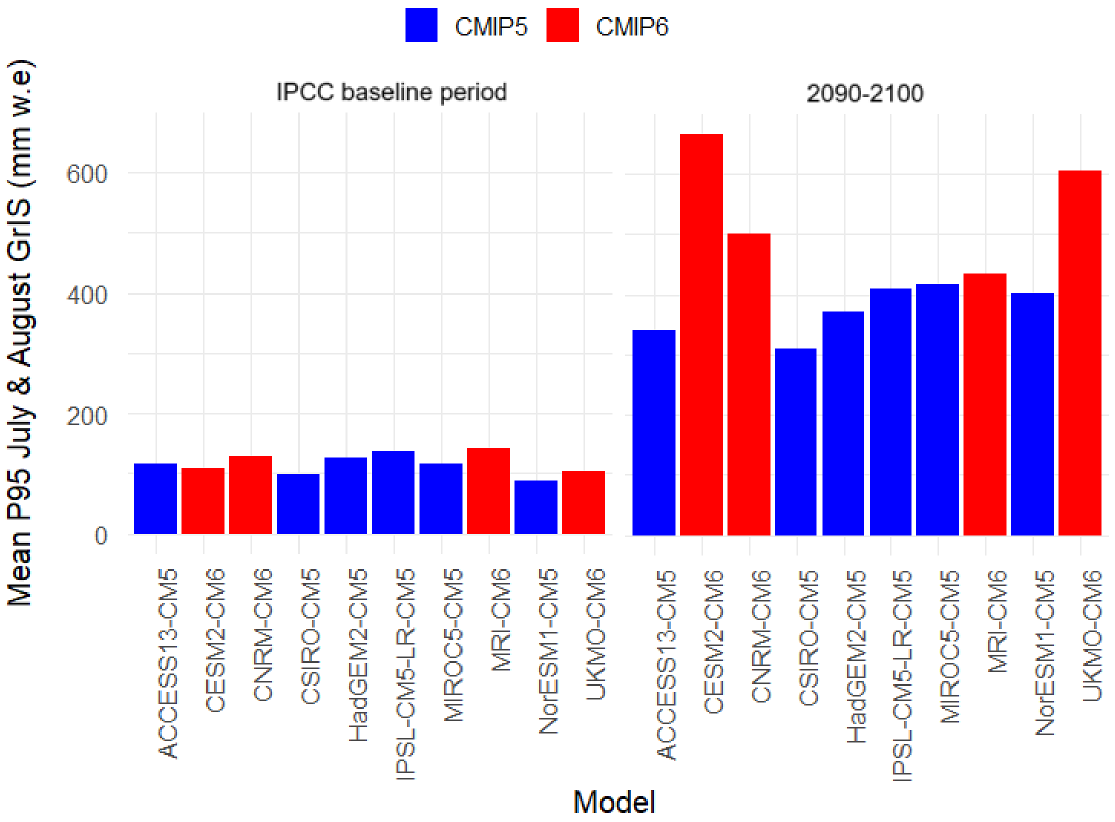

Figure S7. Average monthly 95th percentile of melt over the GrIS in July and August for the IPCC AR6 baseline period (1986–2005, inclusive) and for 2090–2100. Data are shown for each model (x-axis), with CMIP5 and CMIP6 distinguished by color.

Table S1. Summary of the main characteristics of the top 10 extreme melting events analyzed. Note that the event name (Year-Month) corresponds to the end date, with the exception of 2021, where two events occurred with end dates in August. In this case, the first event, which began in July, is labeled as 2021-07.

**Year-Month**	**Start date**	**End date**	**Acc. meltwater event (Gt)**	**Acc. meltwater event detrended (Gt)**	**Peak daily meltwater event (Gt)**	**Peak daily meltwater event detrended (Gt)**	**CWT**
1966-07	1966-07-28	1966-08-05	114,5	60	17,79	11,6	4
1988-07	1988-07-04	1988-07-06	52,1	26,3	19,97	11,8	4
1997-08	1997-08-11	1997-08-15	52	29,1	16,30	11,6	17
2005-07	2005-07-20	2005-07-28	144,6	61,5	20,75	11,2	16
2012-07	2012-07-05	2012-07-20	306,7	141,8	27,8	17,6	17
2012-08	2012-07-26	2012-08-09	261,1	147,8	23,0	16,2	14
2019-07	2019-07-29	2019-08-09	195,4	101,5	29,5	19,5	19
2021-07	2021-07-27	2021-08-01	111,6	53,6	20,28	16,6	20
2021-08	2021-08-13	2021-08-27	135,3	78,6	25,9	14,7	13
2023-08	2023-08-16	2023-08-26	108,4	68,9	19,4	15,8	13

Table S2. Summary of the average meltwater (Gt) from the top 10 extreme melting events and their 10 analog -D events across different time periods. Rows marked as unprecedented indicate no analogs were found within the analyzed period (July and August, 1950–2023).

**Year-Month**	**1950-1975**	**1975-2000**	**1990-2023**	**2000-2023**	**Anomaly (%)**
1966-07	Unprecedented				
1988-07	6,5	7,7	9,2	9,8	33,7
1997-08	5,1	4,6	6,7	6,1	16,4
2005-07	10,7	10,0	13,7	13,7	21,9
2012-07	8,8	8,8	11,0	11,4	22,8
2012-08	Unprecedented				
2019-07	Unprecedented				
2021-07	Unprecedented				
2021-08	4,2	3,3	4,9	5,4	22,2
2023-08	4,5	4,7	6,5	6,9	34,8
